# The Plague

**Published:** 1898-10-29

**Authors:** 


### The Hospital

A Journal of

The Medical Sciences and Hospital Administration.

Vol. XXV.—No. 631.] Edited by Sir Henry Burdett, K.C.B., and Solomon C. Smith, M.D., M.R.C.P. [Oct. 29, 1898.

### The Plague.

It is possible, and, indeed, we think it is probable, that the unfortunate outbreak of plague which has taken place at the pathological institute in Vienna may be a mere flash in the pan, and may lead to no widespread manifestation of the disease. Still, Vienna is so near at hand, is the centre of so large a system of railway communication, and is thus in touch with so vast a population whose social and sanitary surroundings are by no means such as to render them immune to the ravages of the disease if it were once to take root among them, that we cannot but look with some anxiety at what is going on in the Austrian capital.

A few years ago physicians trained in modern medical methods had but little practical knowledge of plague. Isolated outbreaks had during the past fifty years taken place occasionally within the confines of civilisation, but they had quickly died down, and although it was well known that plague still continued to be more or less constantly present in those wild districts which form its endemic home —a belt of territory stretching across mid-Asia— the tendency was to look upon the disease as one which was being gradually beaten back, and was never likely again to intrude into civilised areas. And, no doubt, that view would have been correct had the civilisation, whose progress has been so marked a feature of the century, been more than skin deep. Unfortunately the facilities for social improvement offered by railways, steamships, and other means of communication, and by the far greater productiveness of modern labour, have not always led to any marked advance in the standard of living. The growing thousands have crowded into towns, carrying with them primitive habits fitted only for country life, and thus it has happened that, great as has been the progress of the century in certain directions, it has been accompanied by the growth, here and there all the world over, of immense town populations, living amid conditions of insanitation which would not be safe even for small and isolated communities. It must be remembered also that this great change, and this vast increase of cities, is a comparatively new thing, which has not as yet been through the furnace of experience. Although we have every reason to believe that a true civilisation, one which should extend down to the bottom, and should carry to the lowest social stratum habits of decency and self-control as well as better food and better sanitary surroundings, would take the sting out of most epidemic diseases, we know also that such is not the civilisation of this end of the century, which, indeed, although beautiful to look upon, is decorated with explosive plague spots, liable at any time to lead to disaster. In regard to certain water-borne diseases, such as cholera and typhoid fever, we have undoubtedly made great advances, but in view of the insidious and steady growth which has taken place in such a disease, for example, as diphtheria, notwithstanding all our efforts to control it, we cannot afford to fold our hands and accept as indubitable verities the optimistic hopes of those who say that we are not as other nations, and that plague could never become established within our shores.

Under such circumstances it is of the greatest importance that we should understand from the beginning what are our real protectives against plague, and should nerve ourselves against the popular outcry for quarantine, which would almost certainly arise if the disease became established close at hand. A study of the plague measures which have been current in the affected districts in India, of the extraordinary disturbances to which all trades have been subjected, and of the incredible interferences with liberty which have been enforced wherever the attempt has been made to control the spread of the disease is sufficient to convince anyone of the inapplicabilility of such measures in a country like England. We must not be too sure, however, that the demand would not be made. The United States are held up before us as a model of all that is progressive and intelligent, yet the absurdities which were committed only last year in the Southern States under the influence of the scare about yellow fever were such as are almost incredible in a people of the same race and possessed of the same knowledge as ourselves. When we think of Tommy Atkins going, shovel in hand, into the plague dens of Hong Kong, and passing through the danger, not unscathed, perhaps, but still with but small loss, and when we think of the English nurses and the English medical men who during the height of the Indian epidemic were constantly mixed up with plague, and only in a few instances became affected, we see that direct infection is but one among many factors in the etiology of the disease. It may be that the virus which was in use at Vienna was peculiarly intense, but the teaching of all previous experience is to the effect that the real preventives of plague are cleanliness, fresh air, and good food. Plague is to be fought in the slums; not, however, by the segregation of vast crowds of suspects, but by the immediate removal of those actually affected, by the free use of the scrubbing-brush, and by the amelioration of the conditions of those who dwell in these places.

